# CMOS Current Feedback Operational Amplifier-Based Relaxation Generator for Capacity to Voltage Sensor Interface

**DOI:** 10.3390/s18124488

**Published:** 2018-12-18

**Authors:** Ladislav Polak, Roman Sotner, Jiri Petrzela, Jan Jerabek

**Affiliations:** 1 Department of Radio Electronics, SIX Research Center, Brno University of Technology (BUT), Technicka 3082/12, 616 00 Brno, Czech Republic; polakl@feec.vutbr.cz (L.P.); petrzelj@feec.vutbr.cz (J.P.); 2 Department of Telecommunications, SIX Research Center, Brno University of Technology (BUT), Technicka 3082/12, 616 00 Brno, Czech Republic; jerabekj@feec.vutbr.cz

**Keywords:** capacity sensor interface, capacity measurement, current feedback operational amplifier, relaxation generator, square wave generator

## Abstract

This paper presents a simple relaxation generator, suitable for a sensor interface, operating as a transducer of capacitance to frequency/period. The proposed circuit employs a current feedback operational amplifier, fabricated in I3T25 0.35 μm ON Semiconductor CMOS process, and four passive elements including a grounded capacitor (the sensed parameter). It offers a low-impedance voltage output of the generated square wave. Additional frequency to DC voltage converter offers output information in the form of voltage. The experimental capacitance variation from 6.8 nF to 100 nF yields voltage change in the range from 21 mV to 106 mV with error below 5% and sensitivity 0.912 mV/nF evaluated over the full range of change. These values are in good agreement with simulation results obtained from the Mathcad model of frequency to DC voltage transducer passive circuit.

## 1. Introduction

Electrical sensors form an important part of complex electronic systems, which are used in many fields (e.g., industry, healthcare, consumer electronics and wireless communications) [1]. They are required for transformation of various physical quantities to measurable information in the form of an electrical signal (voltage, current). Such physical quantities, for instance, can be temperature [2], mechanical pressure [3], acoustic pressure [4,5], electromagnetic field [6,7], humidity [8], gas [9,10] and biosignals [11,12]. Due to different operating conditions in the low-voltage (LV) design (e.g., supply voltage and requirement for the power consumption), specific methods and principles of the readout systems must be used. Especially, requirements regarding the LV supply cause restrictions for the implementation of standard methods that are focused on a direct application of quite high voltage levels.

Capacitance sensors enable conversion of various physical changes, for instance, small distance and displacement variation [13,14,15,16] and water level detection [17], to measurable signals. Continuously operating analog interfaces for capacitance sensors use the following methods: (a) AC source-based measurements for sensing of voltage across unknown capacitance and current through unknown capacitance; (b) capacitance divider [18]; (c) resonance [18,19] and bridge circuits containing the measured capacitance [1,18,19]; (d) methods based on the transfer of charge (containing switches and their driving) [19]; (e) differential methods [13,14,15,16] ensuring high accuracy and linearity; and (f) methods based on the sensed capacity as a key part of signal generator [18,19] (sine wave oscillators and generators of other waveforms).

The last method in the previous list can be suitable for LV supply cases, because the signal processing, in comparison with direct measurement of the capacity by applied DC/AC voltage or indirect measurements expecting high voltage levels [15,16], does not depend on the voltage space (voltage levels). Compared to Refs. [15,16], the concept proposed in this paper is simpler. Numerous works dealt with relaxation generators due to their advantages (e.g., low complexity, number of components and cost). Differential methods, in comparison with relaxation generator-based approaches, offer higher accuracy and low measurement error (units of percent). However, these solutions [15,16] are complex. In many cases, these methods evaluate the difference of capacities but not the absolute value of capacitance. Furthermore, various auxiliary components (e.g., control of switching, additional voltage or current sources) are necessary. The expected high output voltage levels [15,16] are not available in LV integrated solutions. Generator-based solutions of transducer are simple because enable direct transformation of capacity to frequency (*C*→*f*${}_{0}$). However, their error is higher (up to 10%).

Table 1 gives an overview of recent works in the field of capacitance interfaces and transducers. Previously proposed concepts are evaluated based on their main features and parameters. Features and advantages of our proposed concept are highlighted in bold. As indicated in Table 1, a dominant part of the proposed solutions uses generator-based methods. In this work, we compared these solutions from the viewpoint of the direct relation to capacitance sensing and transduction. The analysis (see Table 1) led to the following conclusions: (a) the number of the used active elements in many proposed circuits is high [20,21,22,23]; (b) the active device concept employs many sub-parts (3 or 5 current conveyors) [20]; (c) lossless integrator increases the complexity of the proposed concept [20,21,22]; (d) declared measured ranges of “frequency vs. capacitance” dependence are not decisive for the quality evaluation—they always depend on the tested capacitance range (not identical); (e) the output information in the form of DC voltage is unavailable; and (f) the designs have similar inaccuracies (e.g., percentage error, where indicated) in similar ranges [23,24].

It is important to mention the methods that combine analog and digital ways of signal processing for capacitance sensor interfaces [25,26,27,28,29]. The method proposed in Ref. [25] employs controlled charging and discharging of the sensed capacitance and reference capacitance resulting in pulse generation with a variable width in accordance to the sensed capacity value. The methodology in Ref. [26] uses similar approach as Ref. [25], but the reference capacitor is not used here and the output information (the form of a digital word) about the capacity is covered by the changes in the period measured by a counter and processed by a microcontroller. The concept in Ref. [27] implements a phase locked loop evaluating interaction of two ring generators, based on a chain of digital inverters. The first one is controlled by the sensed capacity while the second one is driven digitally. Principle of the capacitance measurement, based on a pressure sensor, is described in Ref. [28]. This method modifies the principle that was previously presented in Ref. [25], but its overall complexity is simpler, because only current source, inverter and subtractor are used. Authors of the work [29] extended and improved a well-known principle that employs two current sources (charging and discharging), and two comparators controlling RS flip-flop. Their improvement consists in the implementation of “ramp and hold” circuit using current flowing through resistor in order to charge the measured capacitor and compare voltages at both elements. From the review of these works, it is evident that all the presented extremely low-power (LP) solutions [25,27,28] target quite a narrow range of the capacity measurement. Unfortunately, in many cases, these devices require an external source of clock or signal for their full operation that significantly increases the power consumption. In summary, principles in the above-discussed papers are totally different from our simple analog proposal (a circuit generates an autonomous waveform) and all the described methods require additional control logic, external clock signal, synchronism and switching accessory. Next, their overall complexity (mixed analog-digital design) and power consumption are significantly higher than our simple analog proposal.

In this paper, a novel concept of capacitance sensor interface is presented, which is based on the well known square wave generator principle. Compared to Ref. [18], where a method employing capacitance divider and calculation from known supplying voltage is used, we propose a solution for implementing two conversions: *C*${}_{\mathrm{sens}}$→*f*${}_{0}$→*V*${}_{\mathrm{sens}}$. Topology of the whole circuitry is simpler than previously proposed concepts; see Refs. [18,20,21,22,23,31,32]. Note that all concepts presented in Refs. [20,21,22,23,24,30,31,32] need an additional *f*${}_{0}$→*V*${}_{\mathrm{sens}}$ converter. Thereby, solutions presented in Refs. [20,21,22,23,31,32] become more complex than concepts presented in Refs. [24,30]. The concept of a two-conversions-based transducer has not been studied and evaluated in these types of generator-based capacitance sensor interfaces.

Compared to state-of-the-art and previously presented solutions (see Table 1), the originality and main contributions of this work are as follows: (a) a new simplified CMOS topology of the active element is proposed and utilized to create a square wave generator with a low impedance voltage mode output; (b) a new simple capacity to voltage sensing interface with low number of passive elements is realized; and (c) an appropriate method to combine of square-wave generator (a *C*${}_{\mathrm{sens}}$→*f*${}_{0}$ converter) and frequency to DC voltage (*f*${}_{0}$→*V*${}_{\mathrm{sens}}$) converter is presented.

Remaining parts of the paper are organized as follows. A new concept of the transducer/interface for the capacitance measurement, its counterparts and their theoretical analysis are described in Section 2. Experimental verification of the established Mathcad model of *f*${}_{0}$→*V*${}_{\mathrm{sens}}$ conversion and results from measurements of the proposed device are presented and compared with theory assumption in Section 3. This section also contains the evaluation of the obtained results. Finally, Section 4 concludes this paper.

## 2. Readout Circuit for Capacity Measurement

**Figure 1:**
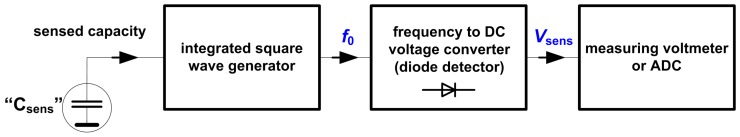
Principle of the capacity measurement.

A block diagram of the capacity measurement, used in the proposed solution, is shown in Figure 1. The sensed capacitor directly determines the oscillation (repeating) frequency, marked as *f*${}_{0}$, of the square wave generator. After that, the frequency is transformed to the DC voltage (*V*${}_{\mathrm{sens}}$). Such a form of the output information is very useful because the capacitance can be measured by a low-cost magneto-electric analog voltmeter. The voltmeter has a scale calibrated as capacitance or the DC voltage can be easily processed by any analog-to-digital converter (ADC).

**Figure 2:**
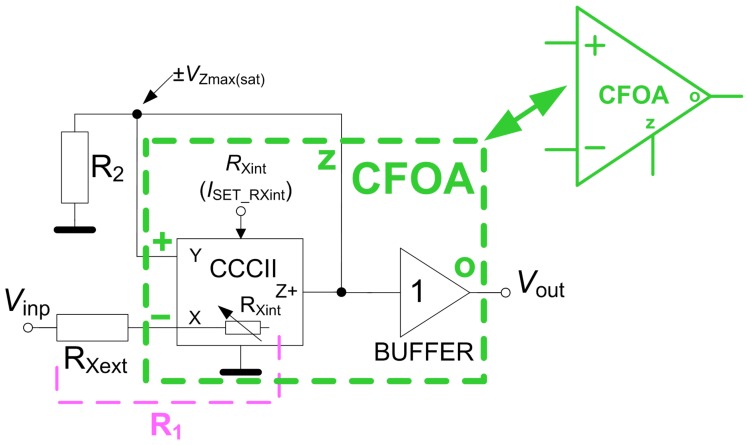
Principle of the proposed comparator.

The integrated square wave generator forms the core of the above briefly described capacity measurement. Its principle is as follows. The generator consists of a special type of Schmitt comparator with hysteresis and an RC network serving as a lossy integrator [1,33]. The RC section is supplied from the output of the comparator (see Figure 2) after impedance separation by a simple voltage follower (buffer). Thanks to this concept (a feedback including buffer), the proposed topology is different from the solution presented in Ref. [23]. Moreover, in Ref. [23], complex active devices are used.

The comparator uses a single current controlled current conveyor of second generation (CCCII) [34,35], a voltage buffer and two resistors. Such an arrangement of active devices is called a current feedback operational amplifier (CFOA) [33]. The principle of CCCII is described by the following inter-terminal relations: *V*${}_{Y}$ = 0, *V*${}_{X}$ = *V*${}_{Y}$ (open X), *V*${}_{X}$ = *V*${}_{Y}$ + *R*${}_{X}$*I*${}_{X}$ and *I*${}_{Z}$ = *I*${}_{X}$. The *V*${}_{o}$ = *V*${}_{z}$ relation is added by the voltage buffer.

The operation of the comparator especially employs the *I*${}_{Z}$ = *I*${}_{X}$ relation. The current, flowing to the X terminal, is directly copied to the Z terminal. It is valid that *I*${}_{R_{2}}$ = *V*${}_{\mathrm{out}}$/*R*${}_{2}$ = ±*V*${}_{\mathrm{Zmax}(\mathrm{sat})}$/*R*${}_{2}$. The saturation voltage of the output Z almost reaches the supply voltage, equals to ±1.65 V. When we consider a postive feedback to the Y terminal and relation between Y and X terminals, where X terminal is terminated by the resistor *R*${}_{1}$ = *R*${}_{\mathrm{Xext}}$ + *R*${}_{\mathrm{Xint}}$ and the voltage *V*${}_{\mathrm{inp}}$ is present at this node, then relation *I*${}_{Z}$ = *I*${}_{X}$ leads to the following expression:

(1) $$\frac{V_{\mathrm{Zmax}(\mathrm{sat})}-V_{\mathrm{inp}(\mathrm{ref})}}{R_{1}}=\frac{V_{\mathrm{Zmax}(\mathrm{sat})}}{R_{2}}.$$

Rearrangement of (1) gives a direct relation for the input threshold symmetrical voltages:

(2) $$\pm V_{\mathrm{inp}(\mathrm{ref})}=\mp V_{\mathrm{Zmax}(\mathrm{sat})}\;\times \left(1-\frac{R_{1}}{R_{2}}\right).$$

**Figure 3:**
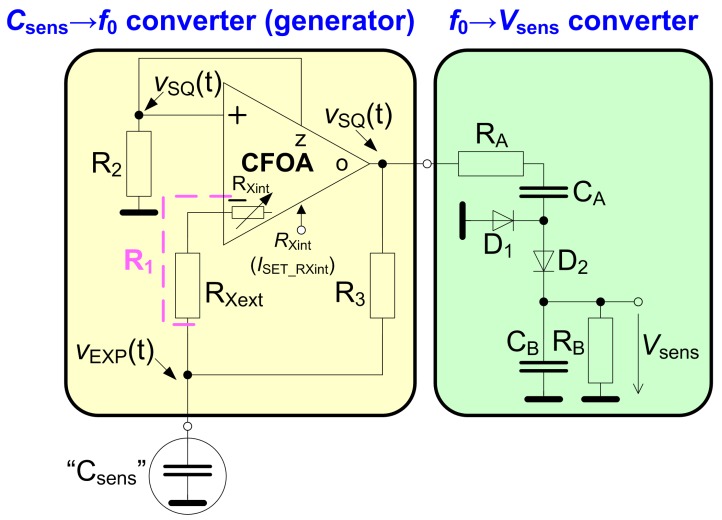
The complete circuitry of the proposed capacity to voltage sensing interface (readout).

The complete circuitry of the capacity to voltage sensing readout is captured in Figure 3. This circuitry consists of two main blocks, namely *C*${}_{\mathrm{sens}}$→*f*${}_{0}$ and *f*${}_{0}$→*V*${}_{\mathrm{sens}}$ converters.

### 2.1. The C${}_{\mathrm{sens}}$→f_0_ Converter

**Figure 4:**
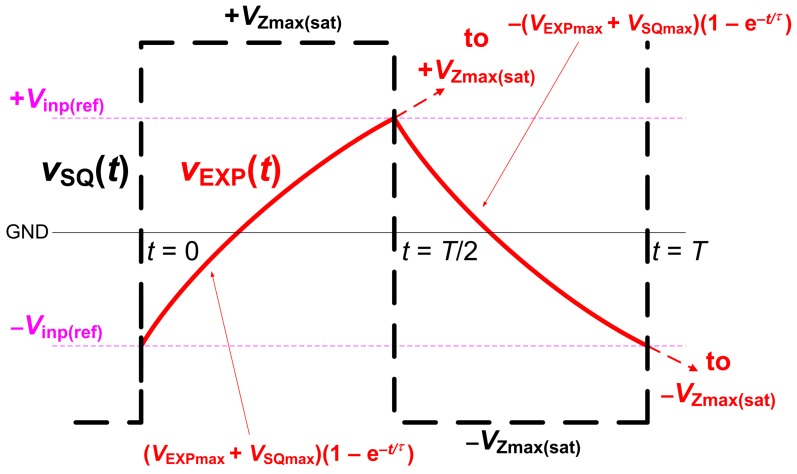
Time diagram of transient responses for analysis of the generator operation.

The *C*${}_{\mathrm{sens}}$→*f*${}_{0}$ converter (a square wave generator) is obtained, when the voltage buffer separates the high impedance output node of the comparator (see Figure 2) and an RC network (*R*${}_{3}$, *C*${}_{\mathrm{sens}}$) is connected between the output node and *V*${}_{\mathrm{inp}}$ (to *R*${}_{\mathrm{Xext}}$) of the comparator. The square wave signal at the output of the buffer, marked as *V*${}_{\mathrm{SQ}}$(t), and the signal in the node of *C*${}_{\mathrm{sens}}$, marked as *V*${}_{\mathrm{EXP}}$(t), are important for further explanation (see time diagram in Figure 4).

The time-constant of the *C*${}_{\mathrm{sens}}$ charging can be expressed as a parallel combination of resistors *R*${}_{1}$ and *R*${}_{3}$: τ = [(*R*${}_{1}$*R*${}_{3}$)/($R{}_{1}+R{}_{3}$)] × *C*${}_{\mathrm{sens}}$. The half period of the charging interval is defined as:

(3) $$V_{\mathrm{EXP}}\left(0\leq t\leq T/2\right)=\left(V_{\mathrm{EXPmax}}+V_{\mathrm{SQmax}}\right)\;\left[1-e^{-\frac{t}{\tau }}\right],$$

where *V*${}_{\mathrm{EXPmax}}$ = *V*${}_{\mathrm{inp}(\mathrm{ref})}$ (threshold voltage of the comparator) and *V*${}_{\mathrm{SQmax}}$ = *V*${}_{\mathrm{Zmax}(\mathrm{sat})}$ (saturation voltage of the comparator). According to *V*${}_{\mathrm{EXP}}$(*t* = *T*/2) = 2*V*${}_{\mathrm{inp}(\mathrm{ref})}$, where capacitor charges from −*V*${}_{\mathrm{inp}(\mathrm{ref})}$ to +*V*${}_{\mathrm{inp}(\mathrm{ref})}$, the following formula can be write for *t* = *T*/2:

(4) $$2V_{\mathrm{inp}(\mathrm{ref})}=\left(V_{\mathrm{inp}(\mathrm{ref})}+V_{\mathrm{Zmax}(\mathrm{sat})}\right)\;\left[1-e^{-\frac{T}{2\tau }}\right].$$

After rearrangement of (4), the period can be expressed as:

(5) $$T=2\tau \ln\left(\frac{V_{\mathrm{Zmax}(\mathrm{sat})}+V_{\mathrm{inp}(\mathrm{ref})}}{V_{\mathrm{Zmax}(\mathrm{sat})}-V_{\mathrm{inp}(\mathrm{ref})}}\right),$$

where voltages and the time-constant can be substituted by (2) and by the above introduced expressions, respectively. After that, the period and repeating frequency *f*${}_{0}$ can be calculated as:

(6) $$T=\frac{1}{f_{0}}=2C_{\mathrm{sens}}\left(\frac{R_{1}R_{3}}{R_{1}+R_{3}}\right)\;\ln\left(\frac{2R_{2}-R_{1}}{R_{1}}\right).$$

The maximum current levels (magnitudes do not considering the current polarity), passing through the passive elements of the topology, are determined as follows:

(7) $$\left|I_{R_{2\max}}\right|=\frac{\left|V_{\mathrm{Zmax}(\mathrm{sat})}\right|}{R_{2}},$$

(8) $$\left|I_{R_{1,3\max}}\right|=\frac{\left|V_{\mathrm{Zmax}(\mathrm{sat})}\right|+\left|V_{\mathrm{inp}(\mathrm{ref})}\right|}{R_{1,3}},$$

(9) $$\left|I_{C_{\max}}\right|=\left|I_{R_{1,3\max}}\right|+\left|I_{R_{2\max}}\right|.$$

### 2.2. The f_0_→V_sens_ Converter

The *f*${}_{0}$→*V*${}_{\mathrm{sens}}$ converter (see Figure 3) consists of a diode doubler including two diodes, two capacitors and two resistors. In many standard applications [36], such a concept operates as a peak detector. However, from the viewpoint of time-constant values of the floating (τ${}_{A}$ = *R*${}_{A}$*C*${}_{A}$) and grounded (τ${}_{B}$ = *R*${}_{B}$*C*${}_{B}$) segments, our case is different. Description of the simplified operation of this block is presented in the following paragraph.

The *V*${}_{\mathrm{SQ}}$(t) voltage changes immediately between +*V*${}_{\mathrm{Zmax}(\mathrm{sat})}$ and −*V*${}_{\mathrm{Zmax}(\mathrm{sat})}$. The negative polarity of *V*${}_{\mathrm{SQ}}$(t) subsequently charges *C*${}_{A}$ to −*V*${}_{\mathrm{Zmax}(\mathrm{sat})}$. When the *V*${}_{\mathrm{SQ}}$(t) turns to +*V*${}_{\mathrm{Zmax}(\mathrm{sat})}$, then the maximal current through *C*${}_{A}$ for *T*/2 can be obtained as *I*${}_{\mathrm{CAmax}}$ = (2*V*${}_{\mathrm{Zmax}(\mathrm{sat})}$ − *V*${}_{D}$)/*R*${}_{A}$. Here, *V*${}_{D}$ marks the voltage drop across the diode (≈0.7 V) and *R*${}_{A}$ is a resistor used to limit the charging current. The maximal voltage (a change across *C*${}_{A}$) determines the overall charge through one period as follows: *Q*${}_{A}$ = 2(*V*${}_{\mathrm{Zmax}(\mathrm{sat})}$ − *V*${}_{D}$) × *C*${}_{A}$. Due to the change of the polarity of *V*${}_{\mathrm{Zmax}(\mathrm{sat})}$, the charge is moved and accumulated by the grounded segment *C*${}_{B}$. The time-constant of the grounded segment is very high. In this case, it is supposed that τ${}_{A}$<<τ${}_{B}$. Consequently, slight discharging of *C*${}_{B}$ in one period is influenced only by the resistor *R*${}_{B}$. It can be expressed as *i*${}_{\mathrm{RB}}$(t) = d*Q*${}_{B}$(t)/dt ≅ *Q*${}_{B}$/*T*. In fact, *i*${}_{\mathrm{RB}}$(t) is almost constant due to high τ${}_{B}$, thereby, *I*${}_{\mathrm{RB}}$ = *V*${}_{\mathrm{sens}}$/*R*${}_{B}$. In the case of *Q*${}_{A}$ = *Q*${}_{B}$ (charge conservation), the ideal relation between the frequency *f*${}_{0}$ and voltage *V*${}_{\mathrm{sens}}$ will be:

(10) $$V_{\mathrm{sens}}≅2\frac{\left(V_{\mathrm{Zmax}(\mathrm{sat})}-V_{D}\right)R_{B}C_{A}}{T}≅2\left(V_{\mathrm{Zmax}(\mathrm{sat})}-V_{D}\right)R_{B}C_{A}f_{0}.$$

Using (10) and (6), it is possible to obtain the relation between *V*${}_{\mathrm{sens}}$ and *C*${}_{\mathrm{sens}}$:

(11) $$V_{\mathrm{sens}}=\frac{\left(V_{\mathrm{Zmax}(\mathrm{sat})}-V_{D}\right)R_{B}C_{A}}{C_{\mathrm{sens}}\left(\frac{R_{1}R_{3}}{R_{1}+R_{3}}\right)\;\ln\left(\frac{2R_{2}-R_{1}}{R_{1}}\right)}.$$

The limitation of validity of (11) concerns periods shorter than time required to accumulate a charge in the floating segment. Thereby, the output *V*${}_{\mathrm{sens}}$ voltage goes to zero. Restriction of the converter for very large periods, where the floating segment has faster response (short time-constant) than the processed signal, must be taken into account. In this case, the charge in the circuit for *f*${}_{0}$→*V*${}_{\mathrm{sens}}$ conversion is not subsequently accumulated (moved from *C*${}_{A}$ to *C*${}_{B}$) in each period of the input signal. The capacitor *C*${}_{B}$ is charged directly by the input signal whereas, the discharge (τ${}_{B}$) is not fast enough. Thereby, this approach cannot be used in the full frequency range of the signal generated by the relaxation generator circuit. This range of operation depends on the processed frequency and time-constants (τ${}_{A}$, τ${}_{B}$).

## 3. Experimental Verification

**Figure 5:**
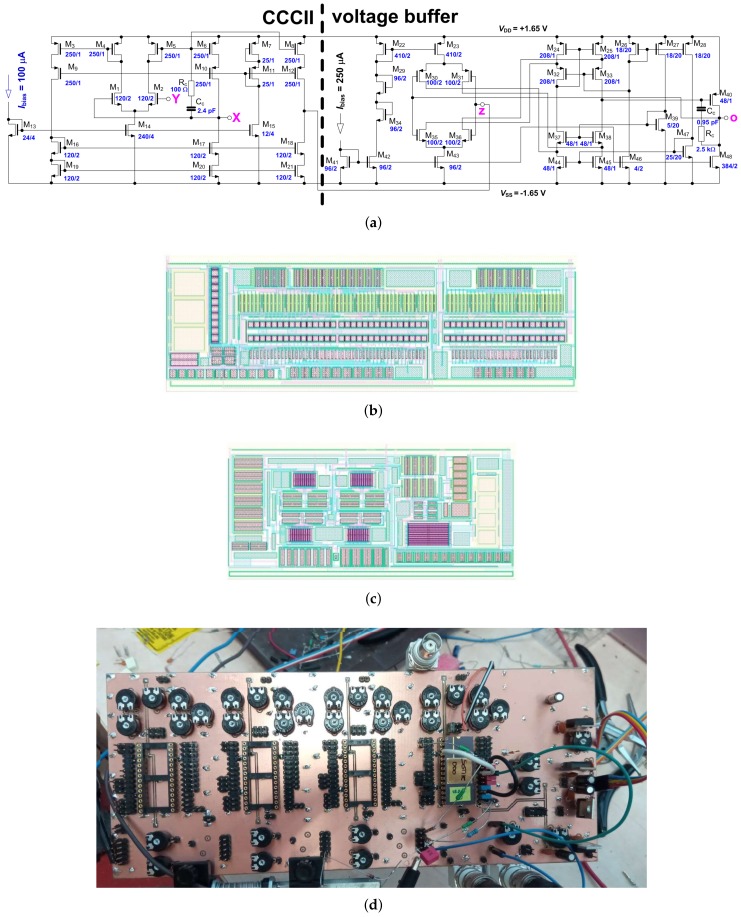
(**a**) The complete CMOS topology of CFOA; layouts of CFOA cells (on a single IC package) fabricated in I3T25 process: (**b**) current controlled current conveyor of second generation (CCCII) and (**c**) voltage buffer; and (**d**) the realized and measured prototype of readout (CFOA and *f*${}_{0}$→*V*${}_{\mathrm{sens}}$ converter).

The complete CMOS topology of the CFOA is shown in Figure 5a. Fabricated cells in ON Semiconductor C035 0.35 μm I3T25 CMOS [37] were used for experimental verification of the proposed concept (see Figure 5b,c). The implementation of CCCII and BUFFER into CFOA element is depicted in Figure 2. The power supply is ±1.65 V and *I*${}_{\mathrm{SETRXint}}$ = 100 μA (*R*${}_{\mathrm{Xint}}$ ≅ 440 Ω). The rest of the external passive elements have the following values: *R*${}_{\mathrm{Xext}}$ = 560 Ω (*R*${}_{1}$ = *R*${}_{\mathrm{Xext}}$ + *R*${}_{\mathrm{Xint}}$ = 1 kΩ), *R*${}_{2}$ = 4.7 kΩ and *R*${}_{3}$ = 1 kΩ. Figure 5d depicts the realized and measured prototype.

The proposed CFOA device has the following features: (a) −3 dB bandwidts > 49 MHz (Y→X), −3 dB bandwidth > 37 MHz (X→z) and −3 dB bandwidth > 45 MHz (z→o); (b) transfers (DC analysis) offer linear processing between ±1 V (for Y→X), ±1.7 mA (for X→z) and ±0.8 V (for z→o); (c) terminal resistances reaches 100 MΩ (Y terminal), >66 kΩ (z terminal), and 280→3400 Ω (X terminal) when internal *R*${}_{X}$ is adjusted by DC bias current from 10 up to 350 μA. Terminal resistance of the o terminal is 0.54 Ω. Parasitic terminal capacities reach values approximately from 2 pF up to 20 pF (it is depending on the design of PCB). The DC input offsets are below 2.5 mV for Y→X transfer, below 6 μA for X→z transfer and below 10 mV for z→o transfer. For the inindicated DC input range, the maximal THD is 1.5% for X→z transfer, 0.6% for Y→X transfer, and 0.5% for z→o transfer.

The values of the passive elements in the *f*${}_{0}$→*V*${}_{\mathrm{sens}}$ converter are as follows: *R*${}_{A}$ = 100 Ω, *C*${}_{A}$ = 1 nF, *R*${}_{B}$ = 1 kΩ and *C*${}_{B}$ = 100 nF. Next, 1N4148 diodes were used. Such values of the passive elements, time-constants as well as parameters in the generator part of the *C*${}_{\mathrm{sens}}$→*f*${}_{0}$ converter are intended to expect capacitance values from units to tens of nF. The value of *V*${}_{\mathrm{Zmax}(\mathrm{sat})}$, equals to ±1.5 V, was obtained from the experiments. We also suppose *V*${}_{D}$ = 0.7 V (standard threshold value for the 1N4148 diode). According to the above considered values, there is predicted a numerical constant from (11) that allows ideal estimation of the relation between the produced DC voltage and the sensed capacity. It can be expressed as *V*${}_{\mathrm{sens}}$ ≅ 8 × 10${}^{-10}$/*C*${}_{\mathrm{sens}}$.

**Figure 6:**
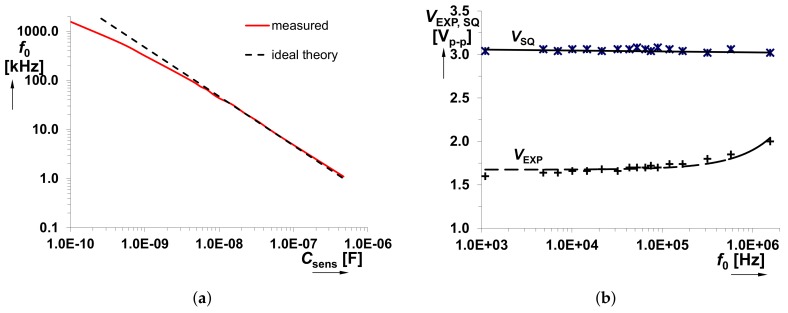
Features of the proposed generator: (**a**) *f*${}_{0}$ versus *C*${}_{\mathrm{sens}}$; (**b**) output levels versus *f*${}_{0}$.

**Figure 7:**
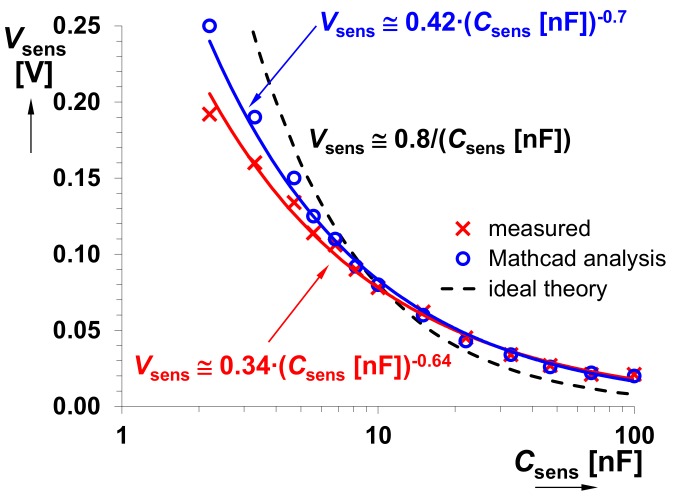
Comparison of the *V*${}_{\mathrm{sens}}$ versus *C*${}_{\mathrm{sens}}$ curves (theory, Mathcad simulations, measurements).

**Figure 8:**
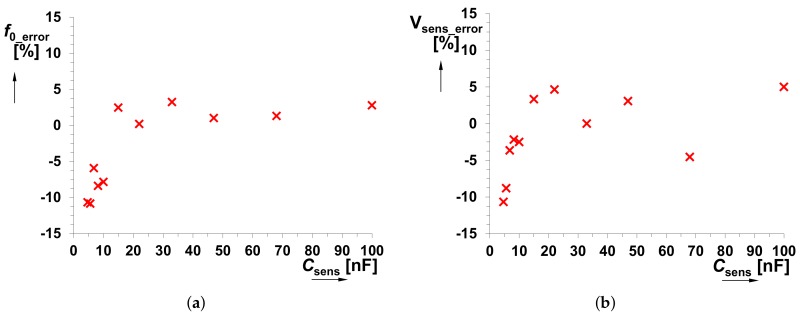
Difference (error) between experimental data and expectation: (**a**) *f*${}_{0\_\mathrm{error}}$ value (theory versus measurement); (**b**) *V*${}_{\mathrm{sens}\_\mathrm{error}}$ value (Mathcad simulation versus measurement).

The *C*${}_{\mathrm{sens}}$ was tested in the range from 100 pF to 470 nF. Dependencies of *f*${}_{0}$ on *C*${}_{\mathrm{sens}}$ and output voltage levels (*V*${}_{\mathrm{SQ}}$ and *V*${}_{\mathrm{EXP}}$) on *f*${}_{0}$ are shown in Figure 6a,b, respectively. A significant influence on the accuracy of the generated *f*${}_{0}$ starts from *C*${}_{\mathrm{sens}}$ < 1 nF. Stability of the square wave output level is also an important feature for the correct operation of the *f*${}_{0}$→*V*${}_{\mathrm{sens}}$ converter. This response is almost constant in the whole operational range of the *f*${}_{0}$ (see Figure 6b). Figure 7 shows the overall system performance, namely *V*${}_{\mathrm{sens}}$ versus *C*${}_{\mathrm{sens}}$. It contains curves obtained from theory, Mathcad calculations and experimental measurements. The complete model of the *f*${}_{0}$→*V*${}_{\mathrm{sens}}$ converter has been implemented in Mathcad in order to verify the correctness of the proposed design. A quite substantial difference is visible between the theory and experimental data. Nevertheless, simulation and measurement results well correlate because the error in the operational range from 6.8 nF to 100 nF is only 5% (see Figure 8). For the case *C*${}_{\mathrm{sens}}$ < 6.8 nF, the difference between them is caused by the inaccuracy of *f*${}_{0}$ (not considered in the Mathcad model). Thereby, theoretical value *V*${}_{\mathrm{sens}}$ ≅ 8 × 10${}^{-10}$/*C*${}_{\mathrm{sens}}$ serves only for orientation purposes.

**Figure 9:**
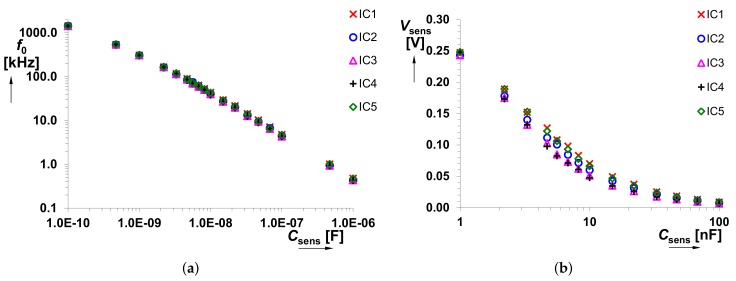
Comparison of the (**a**) *f*${}_{0}$ versus *C*${}_{\mathrm{sens}}$ and (**b**) *V*${}_{\mathrm{sens}}$ versus *C*${}_{\mathrm{sens}}$ for 5 different IC packages (including CFOA device).

**Figure 10:**
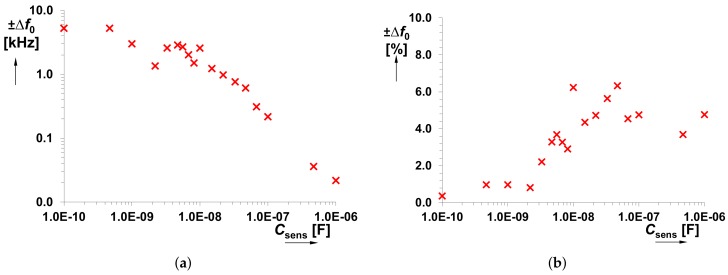
(**a**) Dependence of absolute *f*${}_{0}$ dispersion on *C*${}_{\mathrm{sens}}$ and (**b**) dependence of relative *f*${}_{0}$ dispersion on *C*${}_{\mathrm{sens}}$ caused by fabrication deviation for 5 different IC packages (including CFOA device).

**Figure 11:**
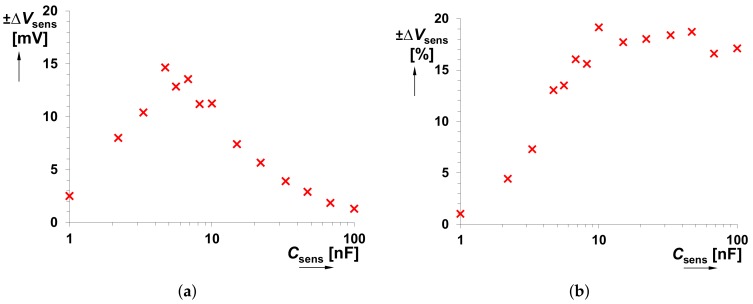
(**a**) Dependence of absolute *V*${}_{\mathrm{sens}}$ dispersion on *C*${}_{\mathrm{sens}}$ and (**b**) dependence of relative *V*${}_{\mathrm{sens}}$ dispersion on *C*${}_{\mathrm{sens}}$ caused by fabrication deviation for 5 different IC packages (including CFOA device).

Figure 9, Figure 10 and Figure 11 capture possible dispersion in the measured *f*${}_{0}$ and *V*${}_{\mathrm{sens}}$ values, when several integrated CFOAs were used. Due to limited amount of available devices, only 5 IC packages were used and tested. Fabrication dispersion causes maximum deviation of *f*${}_{0}$ by ±6% from the nominal value. It means fabrication dispersion of Δ*f*${}_{0}$ = ±5.3 kHz for the lowest value of *C*${}_{\mathrm{sens}}$ and Δ*f*${}_{0}$ = ±22 Hz for the highest value of *C*${}_{\mathrm{sens}}$. The calculated minimal and maximal values for each *C*${}_{\mathrm{sens}}$ are shown in Figure 10. The DC voltage value, obtained at *f*${}_{0}$→*V*${}_{\mathrm{sens}}$ conversion, is influened by fabrication deviation maximally of ±19%. Here, Δ*V*${}_{\mathrm{sens}}$ = ±14.6 mV and Δ*V*${}_{\mathrm{sens}}$ = ±0.15 mV are the minal and maximal values for the considered range of *C*${}_{\mathrm{sens}}$ (0.1 nF→1 μF). It is important to mention that the obtained results (see Figure 10 and Figure 11) are also influenced by the manufacturing dispersion of *C*${}_{\mathrm{sens}}$.

**Figure 12:**
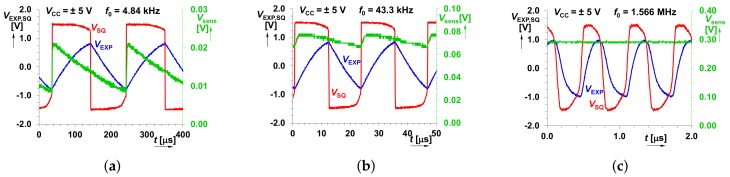
Snapshots of the time domain behavior of the proposed design: (**a**) *C*${}_{\mathrm{sens}}$ = 100 nF (*f*${}_{0}$ = 4.84 kHz, *V*${}_{\mathrm{sens}}$ = 21 mV); (**b**) *C*${}_{\mathrm{sens}}$ = 10 nF (*f*${}_{0}$ = 43.3 kHz, *V*${}_{\mathrm{sens}}$ = 78 mV); (**c**) *C*${}_{\mathrm{sens}}$ = 100 pF (*f*${}_{0}$ = 1 566 kHz, *V*${}_{\mathrm{sens}}$ = 296 mV).

Time domain behavior of the proposed design for *C*${}_{\mathrm{sens}}$ = 100 nF, 10 nF and 100 pF (out of the range of the correct operation) is depicted in Figure 12. The measured *V*${}_{\mathrm{sens}}$ reaches values from 21 mV up to 192 mV for *C*${}_{\mathrm{sens}}$ varied from 100 nF to 2 nF. In the case of simulation, this range is between 20 mV and 250 mV and it is expected at idealized estimation (from 8 mV up to 360 mV). Table 2 summarizes the simulation and measurement results in details.

The minimal detectable capacity (*C*${}_{\mathrm{sens}}$) can be found as a value, where the error between the measured and simulated *V*${}_{\mathrm{sens}}$ values is higher than 10% (see Table 1). This error was studied for the interval <100 pF, 1 μF>. $C{}_{\mathrm{sens}}=5$ nF is the first value where the error is below 10%. Note that, this value can be significantly influenced by the design of the *f*${}_{0}$→*V*${}_{\mathrm{sens}}$ converter and by the parasitic signals influencing the *C*${}_{\mathrm{sens}}$→*f*${}_{0;}$ converter (the connection of *C*${}_{\mathrm{sens}}$). The largest value of *C*${}_{\mathrm{sens}}$ is also limited by time-constants of the inertial character of the *f*${}_{0}$→*V*${}_{\mathrm{sens}}$ converter. In addition, *V*${}_{\mathrm{sens}}$ is rapidly decreasing (units of mV) for the increasing *C*${}_{\mathrm{sens}}$. In our case, the range of the measured *C*${}_{\mathrm{sens}}$ ends at the value of 100 nF, where *V*${}_{\mathrm{sens}}$ reaches acceptable level (more than several units of mV).

## 4. Conclusion Remarks

In this work, a simple concept for capacitance sensing, based on a square wave generator principle, was presented. In addition, the capacitance to frequency transducer has been extended in order to obtain DC voltage by a simple passive converter. Thanks to this modification, the DC output voltage can be easily processed than information obtained at the change of the frequency.

The proposed interface targets a simple interconnection with common low-cost multi-meter or analog voltmeter for fast measurement purposes and simple applications (low-cost measuring device for IC systems where requirements on the range of the sensed capacity and simplicity overweight accuracy). It can be realized by commercially available devices. Due to requirements on the simplicity and very low-power consumption, in this work we have used our previously developed IC device consisting of CCII and voltage buffer cells [38]. Many times, the commercially available AD844 devices consume higher current and require high supply voltage (±15 V). Therefore, the diode detector (*f*→*V* converter) was intentionally supposed as an external part. In addition, for low-frequency design purposes, units of nF up to hundreds of nF are the most commonly used values of the capacitors. Thereby, the designed interface fits these requirements. Next, the proposed concept (active part—integrated CFOA) can be easily implemented as an LP solution consuming a part of a hybrid IC on the PCB. It is important to mention that the interface can be fully integrable in the present form (nF values in external circuit—*C*${}_{\mathrm{sens}}$ only), when the frequency to DC voltage converter part is removed and only the output frequency or period (linear dependence on capacity *C*${}_{\mathrm{sens}}$) are evaluated. The selected values and design specifications are very favorable for the measurement of capacity (units and hundreds of nF), especially in the field of low and medium frequency filter design. Thanks to the LP solution, the interface can be fully integrated when large values of capacities of the frequency to DC voltage converter are replaced by so called capacitance multipliers [34]. Such a solution ensures an appropriate value of the capacity (max. tens of pF) for the integration.

It is possible to have only the frequency as an output signal, but further digital processing is required. Thereby, the simplicity (analog solution) is not fulfilled. In many similar papers, for instance [20,21,22,23,24,30,31,32], only the output frequency of the generator is used as the main sensed information for further processing. However, our proposed approach (measurement of the capacity in the range from untis of nF to hundreds of nF) offers the simplest and low-cost solution for the case when elementary passive topology of the *f*${}_{0}$→*V*${}_{\mathrm{sens}}$ converter is used (see Figure 1 and Figure 3).

The linear processing of the signal has several benefits (see Refs. [16,39]). Our solution, similarly as previous ones, is based on the autonomous signal generation and provides linear dependence of the period on the capacity. The nonlinearity of the DC voltage on the sensed capacity is the cost for the simplicity of the passive converter. On the other hand, it also brings benefit in the form of no additional power consumption (when the *f*${}_{0}$→*V*${}_{\mathrm{sens}}$ converter is replaced by an active device).

The proposed transducer offers an experimentally confirmed range of the capacity measurement starting from 6.8 nF up to 100 nF with error less than 8% for interface producing frequency between 4.8 kHz and 74.8 kHz (sensitivity 7.5 × 10${}^{11}$ Hz/F = 0.751 kHz/nF over a full range of change). Next, it offers the measurment of DC voltage levels from 21 mV up to 106 mV (sensitivity 912 × 10${}^{3}$ V/F = 0.912 mV/nF) with error less than 5%. In addition, the available *R*${}_{\mathrm{Xint}}$ (readjustment by $I_{\text{SET\_RXint}}$) can be used for slight correction of the operation of the proposed concept. To the best of author’s knowledge, such a solution for the capacitance to frequency interface has not been proposed and experimentally verified in previous presented works.

According to the previously presented state-of-the-art, in the specified group of the operation/principle of the square wave generator-based sensing systems, our proposed solution excels mainly with its simplicity and LP consumption. Compared to our proposed concept, previously presented solutions [24,30,31,32] have similar complexity, but they are not enable to deliver output information in the form of DC voltage. In addition, previous solutions do not using up to date compact CMOS active devices. They are based only on the bipolar high-power CFOA structure (commercially available AD844 device) [20,21,22,23,24,31,32]. The number of the used active elements in previous solutions and our solution is comparable (or even higher [20,21,22,23]). Next, previously proposed solutions do not have an option to generate output DC voltage in dependence on the measured capacity value. From the viewpoint of LP performance, previously presented generator-based solutions [20,21,22,23,24,30,31,32] require high supply voltage (>±5 V). This disadvantage in combination with bipolar AD844 leads to large power consumption (hundreds of mW). The power consumption of our proposed concept is only 38 mW.

## Figures and Tables

**Table 1:** Comparison of analog continuous-time operating capacitive sensor interfaces from recent literature (our work is indicated with bold fonts).

References	Number of Active Elements	Type of Active Elements	Principle of Operation	Number of Elements (Grounded/Floating)	Type of Parameter Elements	Waveform at *C*	Range of Sensed Parameter	Number of Decades	Range of Read Parameter	Verification (Real Implementation of Active Device)	Integrated Solution of the Whole System	Supply Voltage	Error	Sensitivity = $\Delta_{f_{0}}$/$\Delta_{\mathrm{capacity}}$ Sensitivity = $\Delta_{V}$/$\Delta_{C}$
Differential methods														
[14]	(b)	(b)	(b)	6 (4/2)	Absolute difference of Cap	-	−0.9 pF→+0.9 pF	-	−0.12 V→+0.12 V	M (CMOS)	Yes	2.5 V	±1.5%	1.33 × 10${}^{11}$ V/F
[15]	4	CCII	I ${}^{\left(a\right)}$	5 (2/3)	Relative difference of Cap	-	−30%→+30%	-	2 V→11 V	M (4×AD844)	No	±10 V	<3%	7.54 × 10${}^{-6}$ V/m
[15]	4	CCII	I ${}^{\left(a\right)}$	5 (2/3)	Relative difference of Cap	-	−30%→+30%	-	0.4 V→2 V	S (CMOS)	partially ${}^{\left(f\right)}$	±1.65 V	<0.23%	1.44 × 10${}^{-6}$ V/m
[16]	4	MLT, OA	II ${}^{\left(a\right)}$	8 (3/5)	Relative difference of Cap	-	−100%→+100%	-	−10 V→+10 V	M (AD844, AD633, INA128, LF411)	No	N/A	±0.8%	N/A
Square waveform generating circuit-based methods														
[30]	1	CCII	III	3 (2/1)	Cap	E	100 pF→700 pF	<1	441 kHz→346 kHz	M (AD844)	No	±10 V	N/A	1.6 × 10${}^{14}$ Hz/F
[20]	3	CCII	III	6 (5/1)	Cap	T	500 pF→5 μF	4	150 kHz→15 Hz	B (CMOS, 3×AD844)	No	±6 V	N/A	3 × 10${}^{10}$ Hz/F
[31]	1	DO-DVCC	III	3 (3/0)	Cap	T	125 pF→10 nF	<2	800 kHz→10 kHz	M (5×AD844)	No	±10 V	N/A	8 × 10${}^{13}$ Hz/F
[32]	1	DVCC	III	3 (2/1)	Cap	E	1 nF→1 μF	3	10 kHz→10 Hz	M (3×AD844)	No	±15 V	N/A	1 × 10${}^{10}$ Hz/F
[21]	2	CCII	III	5 (4/1) ${}^{\left(c\right)}$	Cap	T	10 nF→20 μF	<4	410 kHz→260 Hz	M (2×AD844)	No	N/A ${}^{\left(e\right)}$	N/A	2.1 × 10${}^{10}$ Hz/F
[22]	2	CCII	III	5 (1/4)	Cap	T	0.5 nF→10 μF	<5	263 kHz→25 Hz	M (2×AD844)	No	(±5–±15) V	<5%	2.6 × 10${}^{10}$ Hz/F
[24]	1	CCII	III	4 (1/3)	Cap	E	100 pF→5.5 μF	<5	6.9 kHz→0.14 Hz	M (AD844)	No	N/A ${}^{\left(e\right)}$	≤±10%	1.3 × 10${}^{9}$ Hz/F
[24]	1	CCII	III	4 (1/3)	Cap	E	22 pF→5.5 μF	<6	232 kHz→1 Hz	S (CMOS)	partially ${}^{\left(f\right)}$	±1.2 V	≤±10%	4.2 × 10${}^{10}$ Hz/F
[23]	2	CCII	III	4 (3/1)	Cap	T	500 pF→200 nF	<3	39 kHz→98 Hz	M (2×AD844)	No	±9 V	<7%	2 × 10${}^{11}$ Hz/F
**Proposed (*C*→*f*** ${}_{0}$ **conversion)**														
**This work**	**1**	**CFOA**	III	4 (2/2)	Cap	E	**4.7 nF→470 nF** **6.8 nF→100 nF**	**3** **<2**	**89.3 kHz→1.1 kHz** **74.8 kHz→4.8 kHz**	M (CMOS CCCII, buffer)	partially ${}^{\left(f\right)}$	**±1.65 V**	**<±11%** **<±6%**	**1.9 × 10${}^{11}$ Hz/F** **7.5 × 10${}^{11}$ Hz/F**
**Proposed (*C*→*V* conversion)**														
**This work**	**1**	**CFOA**	III	10 (5/5) ${}^{\left(d\right)}$	Cap	E	**4.7 nF→470 nF** **6.8 nF→100 nF**	**3** **<2**	**0.134 V→0.019 V** **0.106 V→0.021 V**	M (CMOS CCCII, buffer, diodes)	partially ${}^{\left(f\right)}$	**±1.65 V**	**<±12%** **<±5%**	**247 × 10${}^{3}$ V/F** **912 × 10${}^{3}$ V/F**

Notes: I—differential measurement (Δ*C*→*V*), II—bridge balancing (differential measurement of capacity values; Δ*C*→*V*), III—generator (*C*→*f*${}_{0}$), IV—charging and discharging of C and reference capacity and pulse width evaluation, V—period-modulated method, VI—comparison of phases of digitally controlled oscillator and oscillator influenced by capacitance, VII—see discussion in Ref. [29], T—triangular, E—exponential, M—measured, S—simulated, B—both CCII—current conveyor of second generation, CCCII—current controlled current conveyor of second generation, CCII—current conveyor of second generation, CFOA—current feedback operational amplifier, DO-DVCC—differential output—DVCC, DVCC—differential voltage current conveyor of second generation, ENOB—effective number of bits, MLT—multiplier, OA—operational amplifier; ${}^{\left(a\right)}$ external sine wave source required; ${}^{\left(b\right)}$ 3 active devices (transconductance stage, differential and summing current amplifier, switches, buffer, additional DC current sources), sensed difference of switched DC bias currents; ${}^{\left(c\right)}$ 2 capacitors are required; ${}^{\left(d\right)}$ including diodes (Note that column “number of passive elements” includes *C*${}_{\mathrm{sens}}$); ${}^{\left(e\right)}$ supply voltage is not mentioned in the text, but results indicates ±(10–15 V); ${}^{\left(f\right)}$ simulated/measured only at cell level (layout prepared for active device but not shown for fully integrated system).

**Table 2:** Comparison of the obtained results—Mathcad simulations and measurements.

*C*${}_{\mathrm{sens}}$ [nF]	*f*${}_{0(i)}$ [kHz]	*f*${}_{0(m)}$ [kHz]	Error (m versus i) [%]	*V*${}_{\mathrm{EXP}}$ [V]	*V*${}_{\mathrm{SQ}}$ [V]	*V*${}_{\mathrm{sens}(i)}$ [mV]	*V*${}_{\mathrm{sens}(s)}$ [mV]	*V*${}_{\mathrm{sens}(m)}$ [mV]	Error (m versus s) [%]
4.70	100.0	89.3	−10.7	1.70	3.08	170	150	134	−10.7
5.60	83.9	74.8	−10.9	1.72	3.04	143	125	114	−8.8
6.80	69.1	65.0	−5.9	1.70	3.06	118	110	106	−3.6
8.20	57.3	52.5	−8.4	1.70	3.08	97	92	90	−2.2
10.0	47.0	43.3	−7.8	1.70	3.06	79	80	78	−2.5
15.0	31.3	32.1	+2.5	1.66	3.06	53	60	62	+3.3
22.0	21.4	21.4	+0.2	1.68	3.04	36	43	45	+4.7
33.0	14.2	14.7	+3.2	1.66	3.06	24	34	34	0.0
47.0	10.0	10.1	+1.0	1.66	3.04	17	26	27	+3.1
68.0	6.9	7.0	+1.3	1.64	3.06	12	22	21	−4.6
100.0	4.7	4.8	+2.8	1.64	3.04	8	20	21	+5.0
470.0	1.0	1.1	+10.0	1.60	3.06	2	17	19	+11.8

Notes: i—ideal/expected, s—simulation, m—measurement.
